# Mulberroside A ameliorates CCl4‐induced liver fibrosis in mice via inhibiting pro‐inflammatory response

**DOI:** 10.1002/fsn3.3333

**Published:** 2023-03-31

**Authors:** Baozhang Shi, Jinqiang Qian, Hongyue Miao, Shuo Zhang, Yue Hu, Puqing Liu, Liping Xu

**Affiliations:** ^1^ Department of General Surgery Haishu Branch of Ningbo First Hospital Ningbo China; ^2^ Department of Geriatrics Tianjin Medical University General Hospital Tianjin China; ^3^ Department of Hepatobiliary and Pancreatic Surgery Haishu Branch of Ningbo First Hospital Ningbo China; ^4^ Department of Breast Surgery The First Affiliated Hospital of Zhejiang Chinese Medical University Hangzhou China; ^5^ Department of Pharmacy, The Second School of Clinical Medicine Zhejiang Chinese Medical University Hangzhou China

**Keywords:** liver fibrosis, macrophages, Mulberroside A, pro‐inflammatory cytokines

## Abstract

Liver fibrosis is caused by a variety of pathogenic factors. It is mainly characterized by chronic liver damage mediated by the imbalance between extracellular matrix synthesis and degradation. If the injury factor cannot be removed for a long time, fibrosis will progress to cirrhosis or even cancer. The development of liver fibrosis is a very complex process which is related to the activation of hepatic stellate cells (HSCs), oxidative stress, and cytokines produced by immune cells. At present, screening of substances with anti‐inflammatory activity from natural plant extracts has become a new research focus in the prevention and treatment of liver fibrosis. Mulberry twig is a commonly used traditional Chinese medicine. Pharmacological studies have shown that mulberry twig has anti‐inflammatory and antioxidant activities. Thus, it is likely that Mulberry twig contains active substances with liver protection functions. The present study aimed to explore the effect of Mulberroside A (MulA), the main active ingredient from Mulberry twig, on acute liver injury induced by CCl_4_ in mice. MulA treatment could significantly alleviate the CCl_4_‐induced liver injury, as evidenced by histological analysis and Masson staining. However, we observed that MulA inhibited the expressions of collagen I and α‐SMA in livers of CCl_4_‐treated mice but did not directly inhibit the proliferation and activation of HSCs. Finally, we analyzed the anti‐inflammatory effect of MulA and demonstrated that it could markedly inhibit the pro‐inflammatory cytokines release in liver tissues and in cultured macrophages, thereby alleviating liver fibrosis. Our findings suggest MulA as a potential therapeutic candidate for liver injury and inflammatory diseases.

## Graphical abstract

Mulberroside A had no direct effect on HSCs, and it could inhibit the activation of macrophages, which leads to reduced pro‐inflammatory cytokines and may prevent the development of hepatic fibrosis.

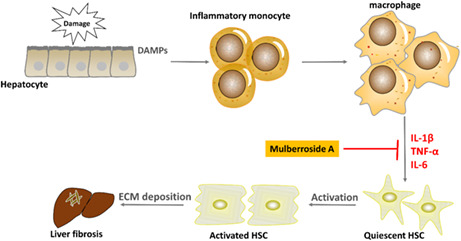

## 1 INTRODUCTION

Liver fibrosis is the wound healing process of liver in response to external stimuli. It involves complex multicellular responses and is often induced by chronic liver injury (Friedman, 2008). A common outcome of progressive liver fibrosis is liver cirrhosis, and China accounts for 11% of global liver cirrhosis‐related deaths (Asrani et al., 2019). Most domestic liver diseases are caused by heavy drinking and chronic hepatitis B or C infection (Liang, 2009; Novo‐Veleiro et al., 2016). No matter what the cause is, the formation of liver fibrosis is very similar, mainly including long‐term chronic parenchymal damage, continuous activation of inflammatory response and oxidation, massive deposition of extracellular matrix, and formation of fibrous scarring, which together destroy the normal structure and function of the liver (Czaja & Carpenter, 2004; Mallet et al., 2008). It is currently believed that the progress of liver fibrosis is irreversible. Thus, there are only a limited number of therapies that can be used to treat fibrosis diseases (Henderson et al., 2020). The use of anti‐fibrosis drugs or the removal of pathogens might be effective measures to treat liver fibrosis. But for patients whose disease has progressed to cirrhosis, liver transplantation is currently the only treatment. Therefore, it is necessary to develop new therapies to treat liver fibrosis (Bataller & Brenner, 2005; Henderson et al., 2020).

Following liver injury, the death of hepatocytes and the infiltration of immune cells lead to a cascade of inflammatory and fibrotic signaling activation (Caligiuri et al., 2021). The damaged cells can release damage‐associated molecular patterns (DAMPs), such as cell‐free DNA, ATP, high mobility group protein B1 (HMGB1), heat shock proteins (An et al., 2020); and the pathogen‐associated molecular patterns (PAMPS) may also be released, such as alcohol and Gram‐negative bacteria lipopolysaccharide (LPS). These patterns stimulate and activate inflammatory cells such as macrophages, T cells, natural killer T cells (NKT), Kupffer cells, which secrete pro‐inflammatory factors such as interleukin (IL)‐1β, IL‐18, IL‐13, interferon γ, and tumor necrosis factor‐α (TNF‐α) (Caligiuri et al., 2021; Nati et al., 2022). In the inflammatory microenvironment, hepatic stellate cells (HSCs) are activated and proliferate. Under the stimulation of chemokines, HSCs migrate and transdifferentiate into myofibroblasts, which is the main cell type that secrete extracellular matrix (ECM) proteins and plays an important role in the progression of liver fibrosis (Czaja & Carpenter, 2004; Liang, 2009). In addition, the role of hepatic macrophages in the occurrence and development of liver fibrosis has become a focus of liver disease research. Pro‐inflammatory macrophages can secrete a variety of inflammatory cytokines to aggravate fibrosis (Cheng et al., 2021; Tacke & Zimmermann, 2014). Screening for substances with anti‐fibrotic and anti‐inflammatory activities from natural plant extracts has also become a new direction in the prevention and treatment of liver fibrosis.

Mulberry twig is a commonly used traditional Chinese medicine (Sakai & Otsuka, 1967). The ancient medical book “The Newly Revise Materia Medica” records that mulberry twig “has a clear and bitter taste, slightly cold, non‐toxic, and enters the liver meridian”. “Suixiju Diet Spectrum” records that mulberry twig can “nourish the liver and kidney, enrich blood”. Contemporary pharmacological studies have shown that mulberry twigs have anti‐inflammatory, antioxidant and other biological activities (Chen et al., 2014; Kim et al., 2021; Novo & Parola, 2008; Qu et al., 2021), and mulberry twigs are likely to contain active substances with good liver‐protection function. However, there is a lack of systematic investigation on the hepatoprotective effect of its main active ingredient Mulberroside A (MulA). In this study, we explored the role and preliminary mechanism of MulA in CCl_4_‐induced liver injury. The results showed that MulA could significantly delay the progress of CCL_4_‐induced liver fibrosis via significantly suppressing the release of pro‐inflammatory cytokines in liver tissues and in cultured macrophages. In summary, our results suggest that MulA could inhibit the activation of macrophages, resulting in reduced pro‐inflammatory cytokines, which may prevent the development of hepatic fibrosis.

## 2 MATERIALS AND METHODS

### 2.1 Chemicals

Mulberroside A (IM0940) was purchased from Solarbio (Shanghai, China). CCl_4_ (C128126) was purchased from Aladdin (Shanghai, China).

### 2.2 Cell culture

Human HSCs LX‐2 was obtained from the Mingzhoubio Company (Ningbo, China) and cultured in RPMI‐1640 Medium (Hyclone). 10% fetal bovine serum (fetal bovine serum (FBS), GIBCO), and 1% streptomycin/penicillin (Shenggong, Shanghai) were added into the growth medium. Murine peritoneal macrophages were collected from C57BL/6 mice on the 4th day after intraperitoneal injection of thioglycollate medium (2 mL, 4% per mouse). The macrophages were cultured in RPMI 1640 Medium supplemented with 10% FBS (Gibco, California, USA) and 100 units/mL streptomycin/penicillin.

### 2.3 Animal study

Six to eight‐week‐old C57BL/c mice were purchased from Shanghai Slac Animal Inc. The mouse experiments followed the institutional guidelines of Haishu Branch of Ningbo First Hospital and the study was approved by the Ethics Committee of Haishu Branch of Ningbo First Hospital. A total of 32 mice were randomly divided into four groups: negative control (Control); CCl_4_ treatment + PBS (phosphate buffer saline, Vehicle); CCl_4_ treatment + MulA (20 mg/g); CCl_4_ treatment + MulA (40 mg/g). To induce liver fibrosis, the mice were intraperitoneally injected with 50% CCl_4_ in olive oil (0.1 mL/100 g body weight) twice a week for 8 weeks. Negative control mice were treated with olive oil only. At the 4th week, 20 or 40 mg/g MulA was intragastrically administered into mice three times a week for 4 weeks. Blood samples were collected to measure the levels of serum aspartate aminotransferase (AST) and alanine transaminase (ALT). Liver tissues were collected for further assays such as histology and Western blot.

### 2.4 Histopathological analysis

Liver pieces were fixed in 10% phosphate‐buffered formalin, embedded in paraffin block, sectioned at 5 μm and stained with hematoxylin–eosin (H&E; Sigma‐Aldrich). Masson's Trichrome stain kit (Fuzhou Maixin Biotechnology Development Co., Ltd.) and Sirius Red stain kit (Jisskang Biotechnology Co., Ltd.) were used to evaluate the degree of liver fibrosis. Briefly, for Sirius Red staining, tissues were fixed in 4% formalin, dehydrated, and embedded in a paraffin block. The paraffin‐embedded liver sections were then deparaffinized, rehydrated, and stained in Sirius red solution for 40 min, followed by hematoxylin‐eosin staining. Next, the sections were rinsed with running water to remove the stain solution, dried, and sealed with neutral quick‐drying glue. For Masson staining, tissues were fixed in 4% formalin, dehydrated, and embedded in paraffin block. Then, the sections were stained with Weigert's iron hematoxylin solution for 5–10 min, rinsed with distilled water for 10 min, and stained with Beibrich scarlet‐acid Fuschin solution for 10–15 min. Next, the sections were differentiated in the phosphomolybdic‐phosphotungstic acid solution for 10–15 min and transferred to aniline blue solution for 5–10 min. Finally, the sections were quickly dehydrated with 95% ethanol and cleared in xylene. After drying, the sections were sealed with neutral quick‐drying glue. Nikon Eclipse TE2000‐S microscope was used for image acquisition. Five images were captured for each section under high magnification field (200 ×).

### 2.5 Quantitative PCR

Total RNA was extracted from LX‐2 cells and liver tissues using RNAiso reagent (Takara, Cat. No. 9109). After DNase I treatment, the RNA was reverse transcribed (Takara, Cat. No. DRR063A), and then qPCR (Takara, Cat. No. DRR041A) was performed on 7500 Real‐Time PCR system (Applied Biosystems, Carlsbad, CA) to measure gene expression levels. The qPCR primers are as follows:

**Table:** 

	Sense‐strand (5′–3′)	Anti‐sense strand (5′–3′)
hCol1α	GATTCCCTGGACCTAAAGGTGC	AGCCTCTCCATCTTTGCCAGCA
hαACTA2	CTGTTCCAGCCATCCTTCAT	TCATGATGCTGTTGTAGGTGG
hGAPDH	GAAGGTGAAGGTCGGAGT	CATGGGTGGAATCATATTGGAA
mCol1α	TAAGGGTCCCCAATGGTGAGA	GGGTCCCTCGACTCCTACAT
mαACTA2	GTGTGAAGAGGAAGACAGCAC	GTGATGATGCCGTGTTCTATCG
mIL‐1β	GAAATGCCACCTTTTGACAGTG	TGGATGCTCTCATCAGGACAG
mTNFα	CGGTGCCTATGTCTCAGCCT	GAGGGTCTGGGCCATAGAAC
mIL‐6	AGTTGCCTTCTTGGGACTGA	TCCACGATTTCCCAGAGAAC
mGAPDH	TGGATTTGGACGCATTGGTC	TTTGCACTGGTACGTGTTGAT

### 2.6 Western blot

To extract proteins, cells were lysed in complete lysis‐M buffer (Roche, Switzerland) and liver tissues were grinded in liquid nitrogen and lysed in complete lysis‐M buffer. The lysates were heated at 90°C for 10 min. Protein concentration was determined by the bicinchoninic acid (BCA) protein assay (Themor, Cat. No. 155209). Equal amount of protein was loaded on SDS–PAGE gel, transferred onto nitrocellulose membrane, and blotted with different antibodies. Anti‐col1α, α‐SMA antibodies was purchased from Abcam Inc. (ab260043, ab5694). Antibodies against MAPK‐p38 pathway were purchased from CST Inc. (9926, 8690). HRP conjugated secondary antibodies were used (Pierce) at 1:10,000, and the membrane was developed using Super Signal West Pico Chemiluminescent substrate (Pierce).

### 2.7 BrdU staining assay

Cells were fixed and treated with DNase, followed by incubation with FITC‐conjugated anti‐BrdU antibody according to instructions (Biolegend, Cat#364103). The flow cytometry assay was performed using a Canto II flow cytometer (BD Bioscience). The data were analyzed using FlowJo v10.0 software (Tree Star).

### 2.8 Statistical analysis

All data are expressed as mean ± SD. The statistical analysis was performed using GraphPadPrism5 software. Student's *t*‐test was used to compare between two groups. *P* < .05 was considered statistically significant, and the significance level was indicated as follows: *, .05 ≥ *p* > .01; **, .01 ≥ *p* > .001; ***, *p* ≤ .001.

## 3 RESULTS

### 3.1 Mulberroside A alleviated CCl_4_ ‐induced liver injury

**FIGURE 1:**
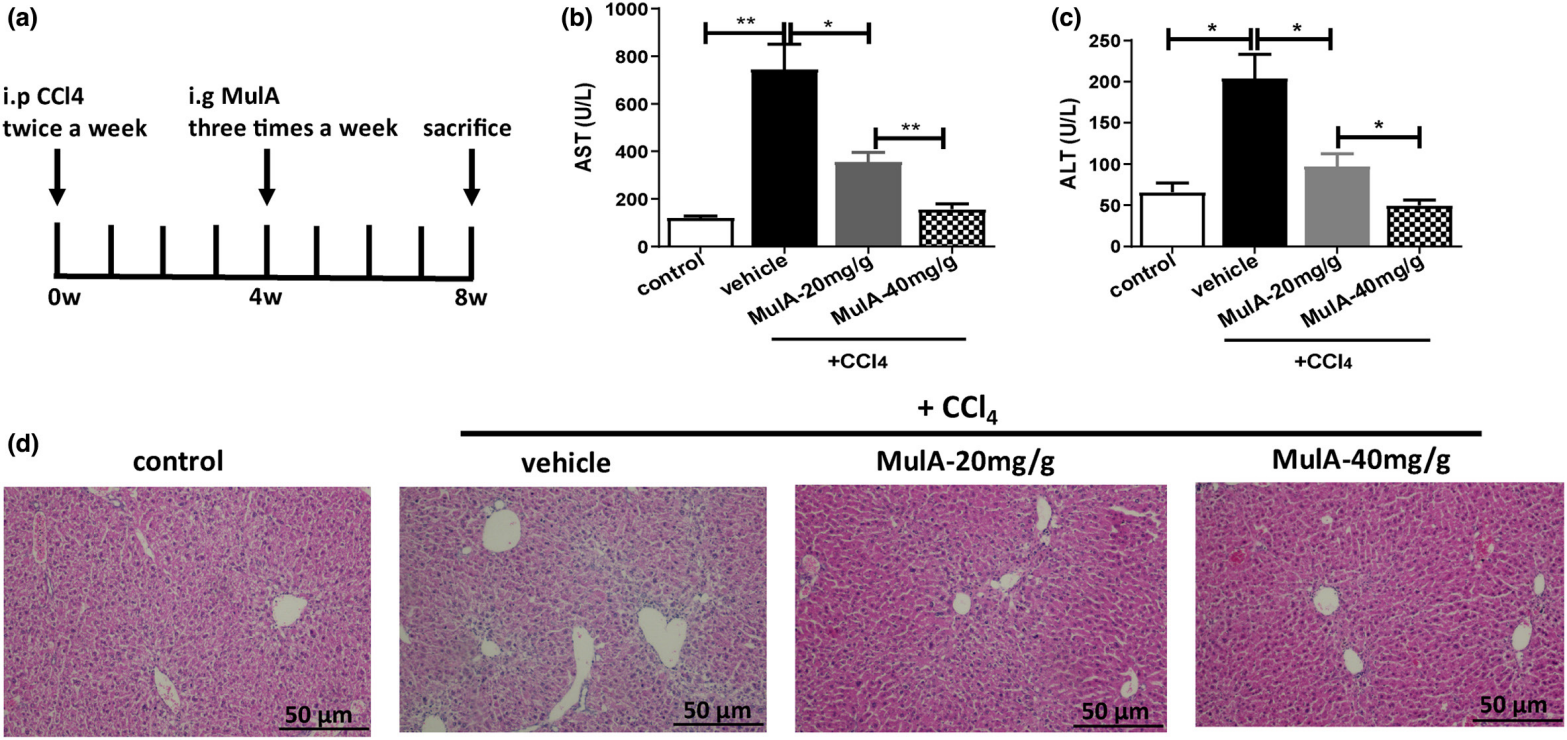
Mulberroside A alleviates CCl_4_‐induced liver injury in mice. (a) Schematic of in vivo experiments. Mice were injected with CCl_4_ twice a week for 8 weeks. At 4th week, mice were treated with PBS or MulA three times a week for the last 4 weeks. (b,c) Hepatic injury was assessed by serum AST and ALT. (d) Liver tissues were subjected to H&E staining for evaluation of necrotic patches and immune cells infiltration.

The mice were intraperitoneally injected with CCl_4_ twice a week and simultaneously received Mulberroside A (MulA) three times a week (Figure 1a). The CCl_4_‐treated mice (experimental group) displayed severe liver damage and increased levels of serum aspartate aminotransferase (AST) and alanine transaminase (ALT), whereas MulA treatment obviously reduced the AST and ALT levels (Figure 1b,c). H&E staining further showed that the CCl_4_‐induced liver damage was significantly improved by MulA treatment (Figure 1d), suggesting that MulA could alleviate the CCl_4_‐induced liver injury.

### 3.2 Mulberroside A inhibited CCl_4_ ‐induced liver fibrosis

**FIGURE 2:**
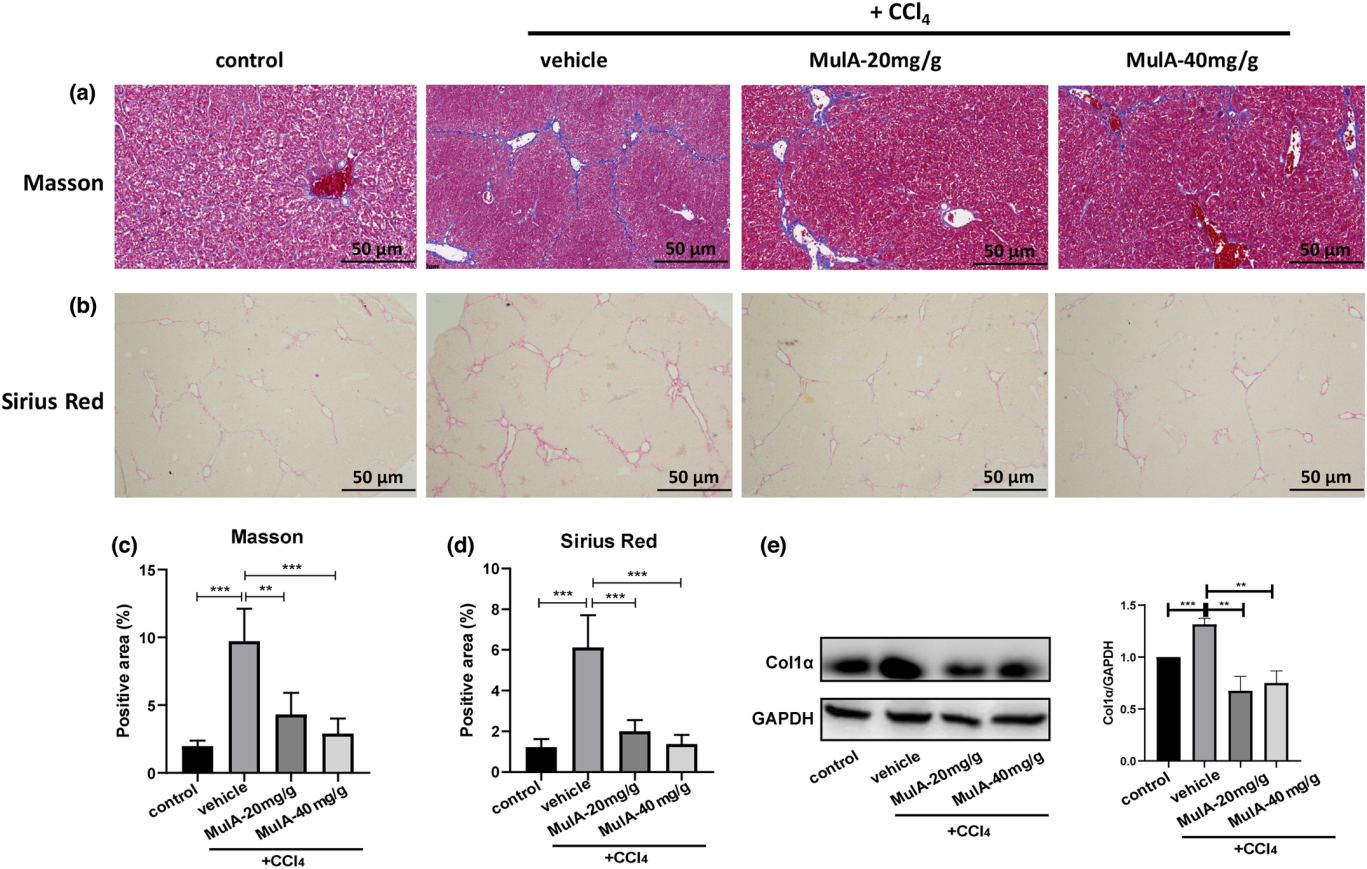
Mulberroside A inhibits CCl_4_‐induced liver fibrosis in mice. Mice liver tissues were subjected to Masson's trichrome staining (a) and Sirius red (b) to analyze the collagen deposition and the proportion of positive area were calculated (c, d). (e) The protein level of Col1α in the liver tissues was detected by Western blot.

**FIGURE 3:**
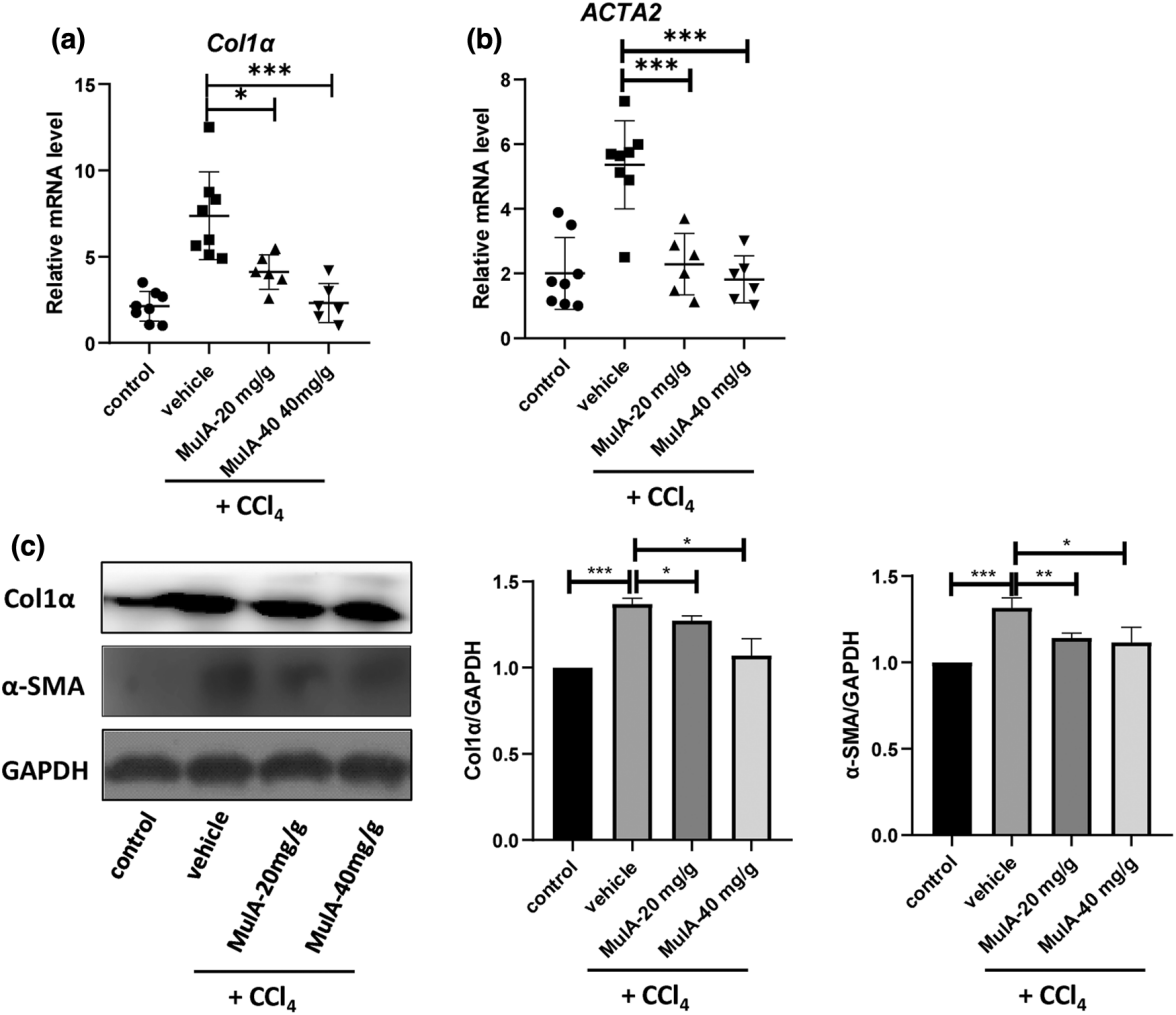
Mulberroside A inhibits the expressions of collagen I and α‐SMA/*ACTA2* in liver tissues. (a, b) Liver tissues were homogenated and subjected to RNA exaction, reverse transcription. Real‐time PCR analyzed the expression of *Col1α* and *ACTA2*. Data are presented as mean ± SD of three representative independents. (c) The protein level of Col1α and α‐SMA were evaluated by Western blot.

Next, we evaluated the degree of liver fibrosis after CCl_4_ injury and MulA administration. Masson's trichrome and Sirius red staining showed that MulA treatment markedly reduced the collagen deposition (Figure 2a–d). Then, we performed Western blot to detect the protein level of Col1 α (collagen, type I, α), and the results showed that MulA treatment could significantly decrease the Col1α protein expression (Figure 2e). Moreover, we measured the expression levels of collagen I and α‐SMA/*ATCT2* genes in liver tissues by qPCR and Western blot. The results showed that the expression levels of collagen I and α‐SMA were significantly suppressed by MulA treatment on both mRNA (Figure 3a,b) and protein levels (Figure 3c). These results consistently demonstrated that MulA could attenuate liver fibrosis in vivo.

### 3.3 Mulberroside A had no effect on HSCs proliferation and activation

**FIGURE 4:**
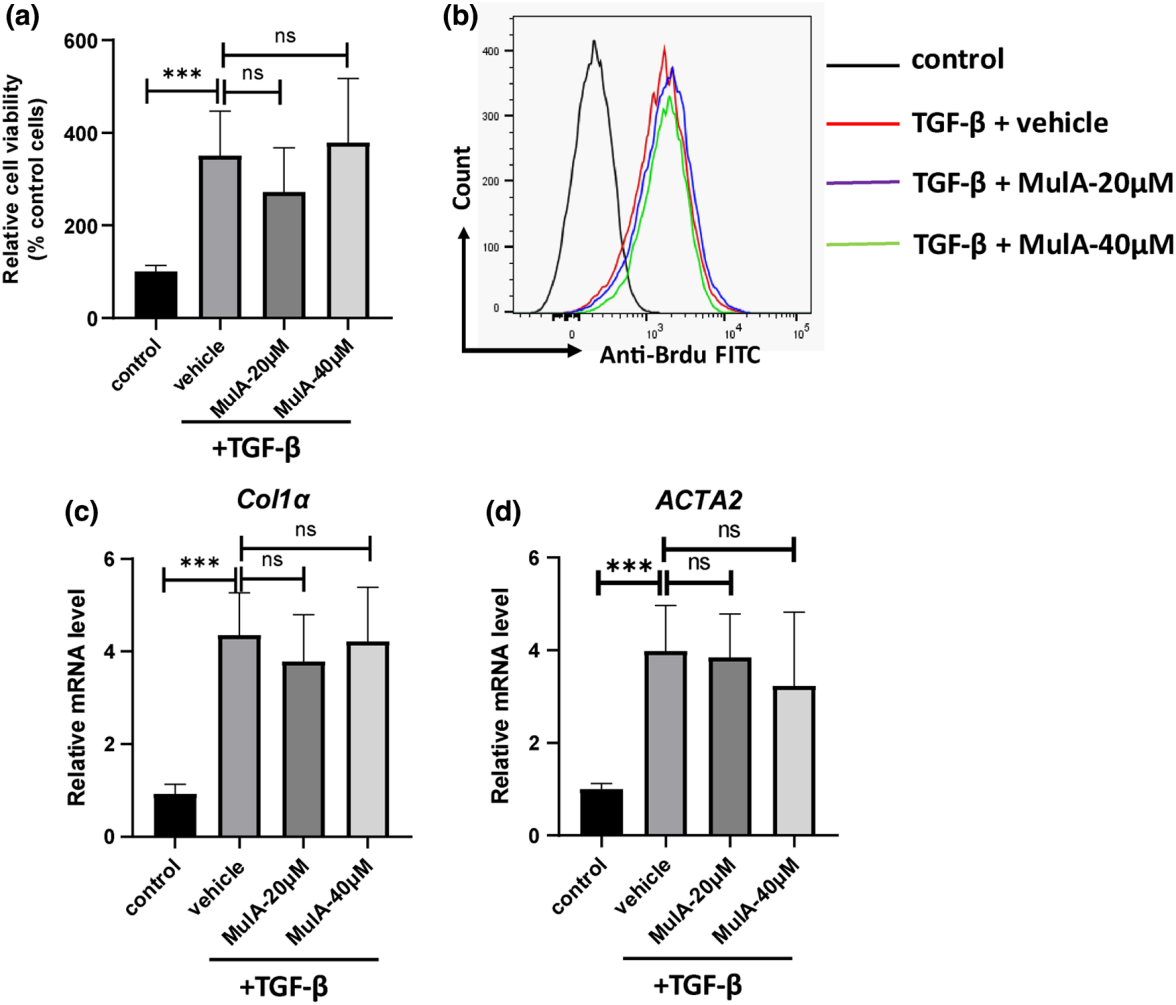
Mulberroside A do not influence LX‐2 cells proliferation and activation. LX‐2 cells were cultured in 24‐well plate and were stimulated with TGF‐β (5 ng/mL) for 24 h. Then, the cells were treated with 20 or 40 μm MulA for 24 h. (a) Cell viability rate was measured by CCK‐8 assay. (b) The proliferation of LX‐2 cells was detected by Brdu staining and analyzed with flow cytometry. (c) The mRNA levels of *Col1α* and *ATCT2* were determined by real‐time‐PCR. Data are presented as mean ± SD of three representative independents.

Considering the key role of HSC activation in promoting the progress of liver fibrosis, we examined the effect of MulA on HSCs proliferation and activation. Firstly, we performed CCK‐8 assay and the result showed that TGF‐β stimulation could significantly enhance LX‐2 cells viability, but MulA treatment had no impact on it (Figure 4a). Then, BrdU assay was performed, demonstrating that MulA treatment did not influence cell proliferation (Figure 4b). Consistently, real‐time PCR further showed that the mRNA levels of collagen I and ACTA2 were not altered by MulA treatment (Figure 4c,d). Thus, these data excluded the possibility of MulA directly influencing HSCs activation.

### 3.4 Mulberroside A suppressed the production of proinflammatory cytokines in vivo

**FIGURE 5:**
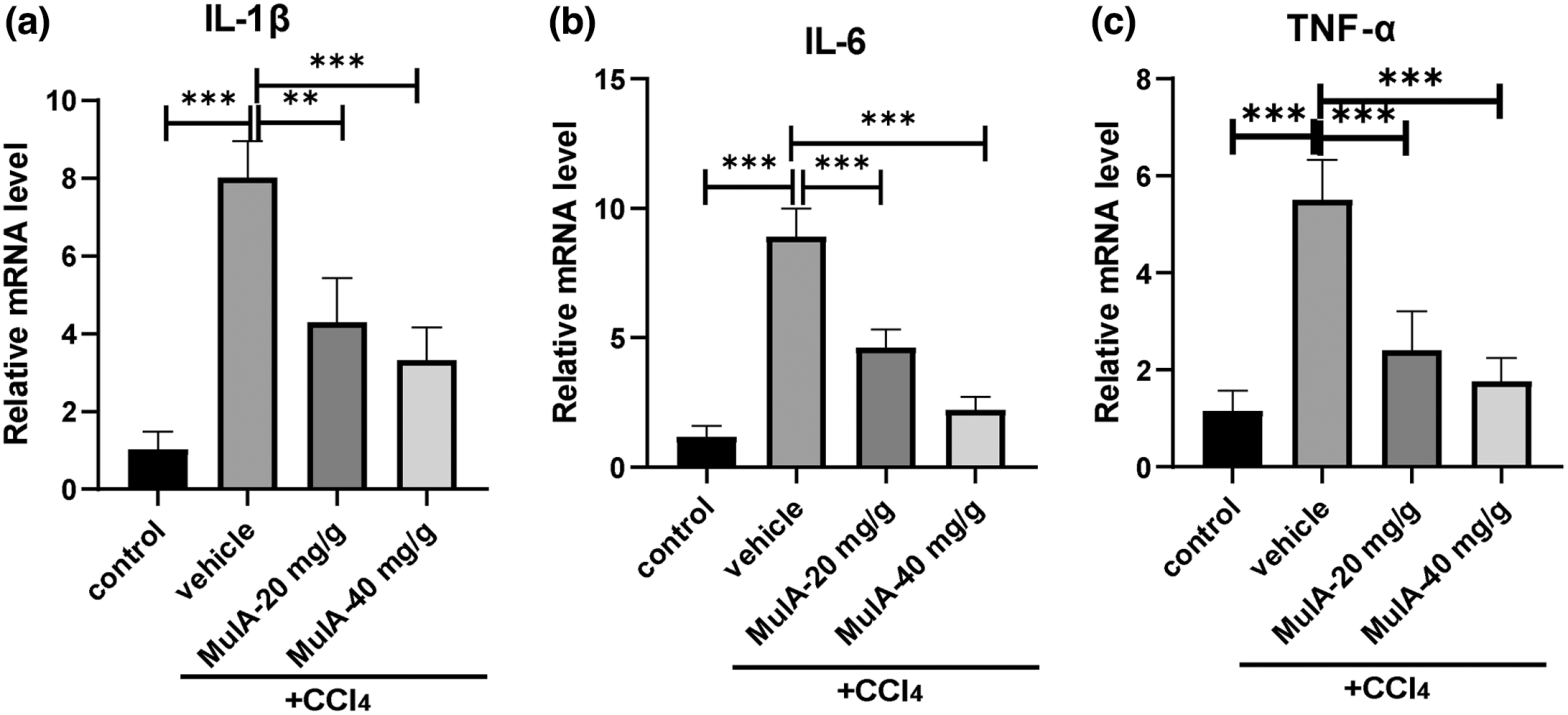
Mulberroside A inhibits the pro‐inflammatory cytokines production in vivo. Liver tissues were homogenated and subjected to RNA exaction, reverse transcription. Real‐time PCR analyzed the expression of TNF‐α, IL‐6 and IL‐1β. Data are presented as mean ± SD of three representative independents.

It has been previously reported that 8 weeks of CCl_4_ challenge could lead to massive infiltration of leucocytes, especially macrophages, which can secrete the pro‐inflammatory cytokines to activate HSCs (Zhao et al., 2017). Therefore, we next examined the effect of MulA on these cytokines in mouse liver tissues. As shown in Figure 5, CCl_4_ treatment could up‐regulate the pro‐inflammatory cytokines TNF‐α, IL‐6 and IL‐1β in the liver, and MulA treatment significantly reduced the levels of these cytokines, indicating that MulA might alleviate liver fibrosis via inhibiting inflammatory cytokines secretion.

### 3.5 Mulberroside A inhibited macrophages activation and indirectly suppressed HSC's activation

**FIGURE 6:**
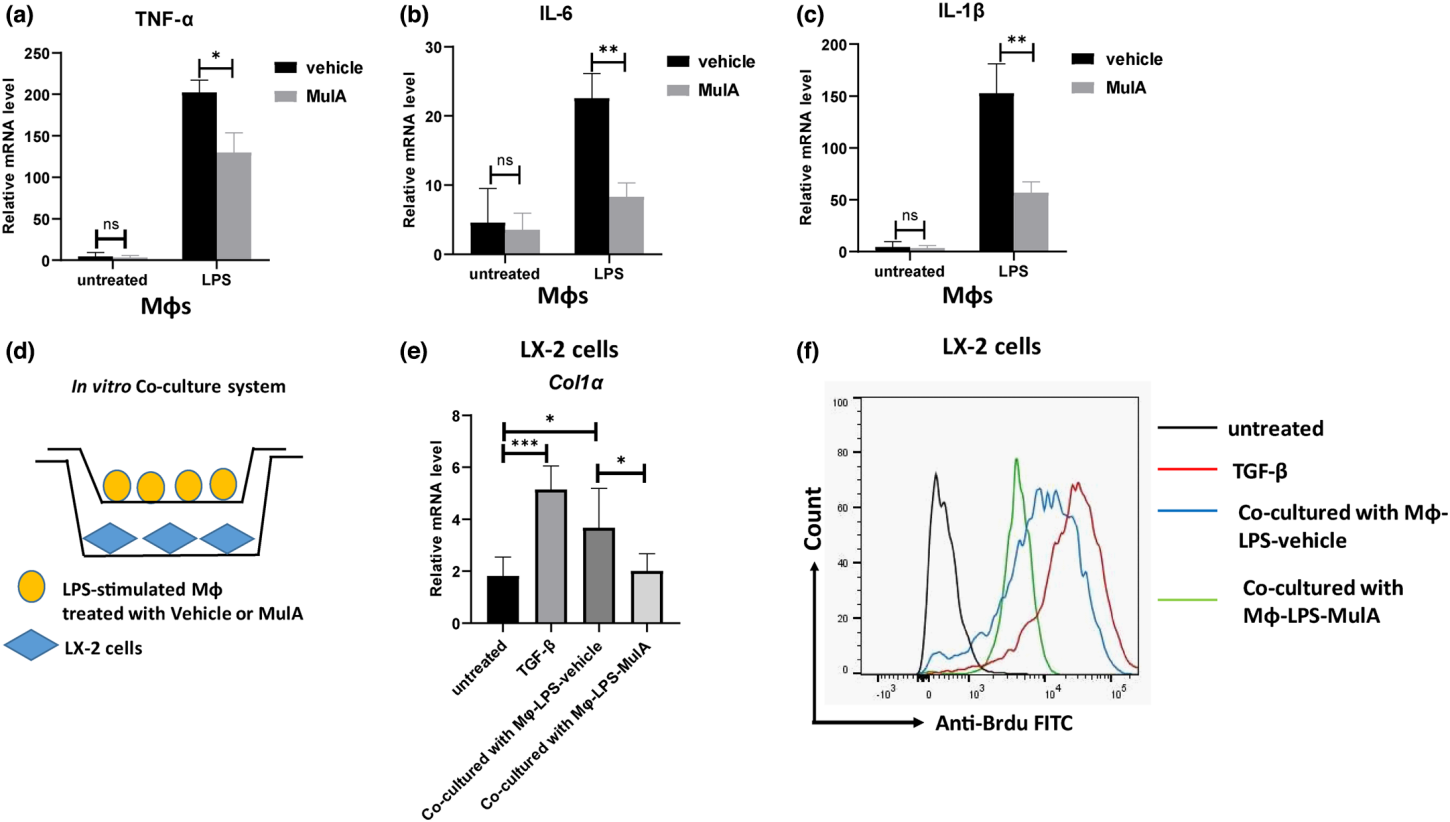
Mulberroside A inhibits macrophages activation. (a–c) Murine peritoneal macrophages were collected from C57BL/6 mice at day 4 post intraperitoneally injecting with thioglycollate medium (2 mL, 4% per mouse) and cultured in 24‐well plate. After stimulating with LPS (100 ng/mL) for 12 h, cells were treated with 20 or 40 μm MulA for 24 h. The mRNA levels of TNF‐α, IL‐6 and IL‐1β were determined by real‐time‐PCR. Data are presented as mean ± SD of three representative independents. (d) Schematic of the co‐culture system. Macrophages were stimulated with LPS (100 ng/mL), treated with MulA, seeded in the top chambers, and co‐cultured with LX‐2 cells, which were seeded in the lower chambers. The cells were co‐cultured for 24 h. (e) LX‐2 cells‐derived Col1a was detected by qPCR. (f) LX‐2 cell proliferation was detected by Brdu staining and analyzed with flow cytometry.

It has been previously shown that MulA can inhibit the activation of MAPK signaling pathway to suppress the pro‐inflammatory response (Jia et al., 2019). To verify it, we isolated primary murine peritoneal macrophages to detect the regulatory effect of MulA on macrophages activation. As shown in Figure 6a–c, MulA treatment significantly decreased the mRNA levels of pro‐inflammatory cytokines TNF‐α, IL‐6 and IL‐1β in macrophages upon LPS stimulation. Then, we co‐cultured LX‐2 cells with macrophages. As shown in Figure 6d, macrophages were stimulated with LPS (100 ng/mL), treated with MulA, seeded on the top chambers, and then co‐cultured with LX‐2 cells, which were seeded in the lower chambers. After 24 h, the cells were harvested for qPCR and BrdU assay. The result showed that MulA‐treated macrophages could significantly decrease the collagen I expression (Figure 6e) and inhibit LX‐2 cells proliferation (Figure 6f). Collectively, these results suggested that MulA might inhibit the inflammatory response of macrophage, resulting in suppressed HSC's activation and improved liver fibrosis.

## 4 DISCUSSION

The traditional Chinese medicine Morus alba is the dry root bark of *Morus alba L*. According to ancient medical books, Morus alba is cold in nature and sweet in taste; it enters the lung and spleen meridians, and has the effects of purging the lung, relieving asthma, reducing swelling, and diuresis. The recent research on Morus alba is making rapid progresses, and a series of compounds have been isolated and identified, mainly including flavonoids, furans, coumarins, terpenes, stilbenes, sterols, sugars, and volatile oils (He et al., 2018). Mulberryside is the main stilbene glycoside active ingredient in Morus alba, mainly including mulberryside A, mulberryside B, mulberryside C, mulberryside D, mulberryside E, mulberryside F, etc. (He et al., 2018). It is mainly used in the treatment of gout, arthritis and rheumatism, and its major role in Chinese medicine is diuresis and reducing edema. In recent years, with the rapid development of molecular biology technologies, extensive studies have been conducted on mulberry and made significant progresses, especially in the pharmacological effects of mulberry, which has become a hot research focus. Both traditional medicine and modern pharmacological studies have shown that mulberry has strong biological activity and various pharmacological effects. In this study, we analyzed the effect of mulberroside A on the CCl_4_‐induced mouse liver fibrosis. The results showed that mulberro A could significantly delay the progress of liver fibrosis in mice.

The liver is an important metabolic organ in the human body. It is the main organ for regulating metabolism, deoxidization, storing glycogen, and synthesizing secreted proteins. The hepatic lobule is the basic structural and functional unit of liver. It is in polygon shape and is mainly composed of the hepatic plate, bile duct, central vein, hepatic sinusoid, and perisinusoidal space (Friedman, 2008). Under normal physiological conditions, there is a layer of low‐density basement membrane that separates blood sinusoids and liver parenchyma, ensuring normal metabolism between different substances. When the liver tissue is damaged, the HSCs in perisinusoidal space are activated and secreted a large amount of ECM, which leads to septum thickening, collagen accumulation, and ultimately liver fibrosis (Tsuchida & Friedman, 2017). In recent years, the research on liver fibrosis has mainly focused on the promotion of HSC apoptosis and the inhibition of HSC activation; PDGF, TGF‐β, hedgehog, and other signaling pathways are all involved in HSC activation. Under normal physiological conditions, HSCs are in a quiescent state. When the liver is stimulated by physical, chemical, and biological factors, quiescent HSCs are transformed into fibroblasts with proliferative and contractile properties. This process is called activation or trans‐differentiation. In the process of activation, HSCs first undergo gene level changes, resulting in increased synthesis and continuous accumulation of ECM, which leads to cell fibrosis; secondly, after activation, the proliferation rate of HSC increases, and the number of fibroblasts in liver tissue also increases (Zhang et al., 2016). The upregulation of α‐SMA is usually considered as a characteristic of myofibroblasts transitioned from activated HSCs, which leads to not only an increased number of HSCs, but also a decreased expression of type 1 collagen (Rockey et al., 2019). So, HSCs are closely related to the formation and development of fibrosis. Therefore, we also aimed to determine whether MulA had a direct effect on HSC activation. However, the results showed that MulA had no effect on the proliferation of HSC cells and the release of profibrotic factors after activation (Figure 4), indicating that it may indirectly regulate the activation of HSC through other pathways.

It has been shown that macrophages play a dual role in the process of fibrosis: some macrophages can inhibit the process of fibrosis, and these cells are called pro‐fibrotic degrading macrophages; while the other macrophages can promote the process of fibrosis, and they are called pro‐fibrotic macrophages (Cheng et al., 2021). Different types of macrophages have different characteristics in surface markers expression and the secretion of cytokines and chemokines. Type I macrophages (M1) are classical macrophages activated by interferon‐γ and bacterial LPS, and they play an important role in promoting the inflammatory response in the early stage of inflammation (Tacke & Zimmermann, 2014; Wu et al., 2020). The cytokines released in the inflammatory response can also promote HSC activation and proliferation, thereby affecting fibrosis. Therefore, we cultured macrophages in vitro and explored the effect of MulA on their activation (Figure 5). Furthermore, we used the co‐cultured system, where LX‐2 cells were co‐cultured with LPS‐primed‐macrophages treated with MulA (Figure 6d). The results showed that the levels of Col1a and Acta2 in LX‐2 cells were obviously decreased when macrophages were treated by MulA (Figure 6e,f). This result may partly support the hypothesis that MulA could inhibit the inflammatory response of macrophage, resulting in suppressed HSC activation and improved liver fibrosis.

In summary, our study explored the role of MulA in regulating liver fibrosis. As shown in Figure S1, although MulA had no direct effect on HSCs, it could inhibit the activation of macrophages, which leads to reduced pro‐inflammatory cytokines and may prevent the development of hepatic fibrosis. Therefore, our findings expand the roles of MulA in hepatic fibrosis and provide novel evidence to support MulA as a potential therapeutic candidate for liver injury and other inflammatory diseases.

## CONFLICT OF INTEREST STATEMENT

The authors declare that they do not have any conflict of interest.

## ETHICS STATEMENT

The mouse experiments followed the institutional guidelines of Haishu Branch of Ningbo First Hospital and the study was approved by the Ethics Committee of Haishu Branch of Ningbo First Hospital.

## INFORMED CONSENT

Written informed consent was obtained from all study participants.

## Supporting information


Figure S1.

## Data Availability

The data that support the findings of this study are openly available.
