# Supplementary material for: Mulberroside A ameliorates CCl4‐induced liver fibrosis in mice via inhibiting pro‐inflammatory response
Source: Food Sci Nutr. 2023 Mar 31;11(6):3433–41. doi: 10.1002/fsn3.3333 (PMC10261818; doi:10.1002/fsn3.3333)
Supplement: Supplementary file 1 — Figure S1. [file FSN3-11-3433-s001.docx]

**
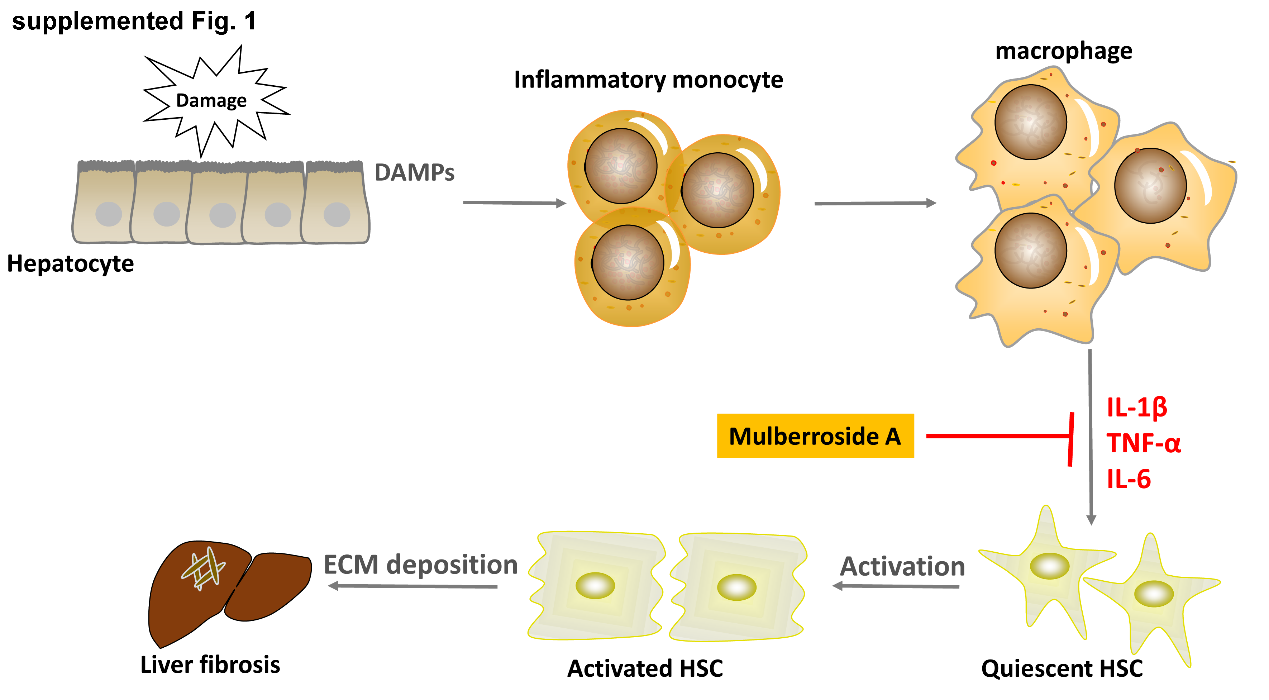
**

**Supplemented Figure 1. The proposed protective role of MulA on the progress of liver fibrosis.**

Mulberrosid A could not directly inhibit HSCs proliferation and activation. However, MulA could inhibit the activation of macrophages and the reduction of proinflammatory cytokines may prevent development of hepatic fibrosis. MulA could inhibit the inflammatory response of macrophages, thus indirectly suppressing HSC activation.
